# Dose-dependent inhibition of gastric injury by hydrogen in alkaline electrolyzed drinking water

**DOI:** 10.1186/1472-6882-14-81

**Published:** 2014-03-03

**Authors:** Jinling Xue, Guodong Shang, Yoshinori Tanaka, Yasuhiro Saihara, Lingyan Hou, Natalia Velasquez, Wenjun Liu, Yun Lu

**Affiliations:** 1 State Key Joint Laboratory of Environmental Simulation and Pollution Control, School of Environment, Tsinghua University, Beijing 100084, P.R. China; 2 State Environmental Protection Key Laboratory of Microorganism Application and Risk Control, Tsinghua University, Beijing 100084, P.R. China; 3 Panasonic Corporation, Appliances Company, 2-3-1-2 Noji-Higashi, Kusatsu-city, Shiga 525-8502, Japan

**Keywords:** Alkaline electrolyzed water, Dose–response, Gastric injury, Hydrogen, Oxidative stress

## Abstract

**Background:**

Hydrogen has been reported to relieve damage in many disease models, and is a potential additive in drinking water to provide protective effects for patients as several clinical studies revealed. However, the absence of a dose–response relationship in the application of hydrogen is puzzling. We attempted to identify the dose–response relationship of hydrogen in alkaline electrolyzed drinking water through the aspirin induced gastric injury model.

**Methods:**

In this study, hydrogen-rich alkaline water was obtained by adding H_2_ to electrolyzed water at one atmosphere pressure. After 2 weeks of drinking, we detected the gastric mucosal damage together with MPO, MDA and 8-OHdG in rat aspirin induced gastric injury model.

**Results:**

Hydrogen-dose dependent inhibition was observed in stomach mucosal. Under pH 8.5, 0.07, 0.22 and 0.84 ppm hydrogen exhibited a high correlation with inhibitory effects showed by erosion area, MPO activity and MDA content in the stomach. Gastric histology also demonstrated the inhibition of damage by hydrogen-rich alkaline water. However, 8-OHdG level in serum did not have significant hydrogen-dose dependent effect. pH 9.5 showed higher but not significant inhibitory response compared with pH 8.5.

**Conclusions:**

Hydrogen is effective in relieving the gastric injury induced by aspirin-HCl, and the inhibitory effect is dose-dependent. The reason behind this may be that hydrogen-rich water directly interacted with the target tissue, while the hydrogen concentration in blood was buffered by liver glycogen, evoking a suppressed dose–response effect. Drinking hydrogen-rich water may protect healthy individuals from gastric damage caused by oxidative stress.

## Background

Endogenous hydrogen is produced by colonic fermentation in the gastrointestinal tract of rodents [1], humans [2], and even insects [3,4]. Hydrogen rapidly penetrates tissues and blood vessels by free diffusion, and then is transported to all organs. The average H_2_ concentration on the mucus layer of the mouse stomach is 43 μM [5]. While in liver, spleen and small intestine, the concentrations are 53, 48, and 168 μM respectively [6].

The physiological role of H_2_ is not yet clear. Ohsawa et al. [7] found that hydrogen has an antioxidant and anti-apoptotic role which protects the brain against ischemia-reperfusion injury and stroke by selectively neutralizing hydroxyl radicals and peroxynitrite. These results therefore suggest that H_2_ can be applied therapeutically as a medical gas. Moreover, clinical application of H_2_ has shown many additional advantages. Firstly, H_2_ does not react with superoxide anion radical and hydrogen peroxide, which have important physiological roles [7]. Secondly, it can be easily delivered via gas, drinking water, and intravenous infusion; and its favorable distribution features allow it to reach many organs that other drugs may not reach, granting it access into the mitochondria, nucleus, and across the blood–brain barrier. And finally, H_2_ elicits little side effects. Its application in Hydreliox, an exotic breathing mixture of 49% hydrogen, 50% helium and 1% oxygen, used in deep diving, demonstrates its safety for human use [8,9]. Likewise, six clinical trials, among which the longest treatment was 6 months, also showed no detectable adverse effects from hydrogen in drinking water, hemodialysis or intravenous infusion [10-16].

In the last five years, the evident protective effects of H_2_ have been documented for 63 disease models and human diseases [17], including cerebral infarction, hepatic and myocardial injury, Parkinson disease, metabolic syndrome, inflammation and allergy, organ transplantation, and so forth [18-20]. However, the possible effects of hydrogen on gastric injury are yet to be studied, and furthermore, the absence of a dose–response effect in the use of hydrogen as a therapeutic molecule in previous studies is surprising for several reasons. First, the amount of hydrogen taken up by drinking water is much less than by inhaling 1-4% hydrogen gas, however hydrogen-rich water showed similar or even better beneficial effects than hydrogen gas [10,21]. Second, the amount of endogenous hydrogen generated by intestinal bacteria (about 1 liter/day) is much more than the amount from drinking hydrogen-rich water (usually less than 50 milliliter/day) [18,21]. Third, drinking hydrogen-rich water in different concentrations, injecting different quantities of hydrogen saline, or inhaling different amounts of hydrogen gas did not show a discernible difference in effects [22-24].

In this study, alkaline hydrogen-rich water was generated through electrolysis. Mixed hydrogen and nitrogen gas (2:8 and 7:3) was used to adjust the hydrogen concentration in water to get three different hydrogen concentrations (0.07 ppm, 0.22 ppm, 0.84 ppm). We did not perform neutralization as most studies did in the past [25-28], since both high pH and hydrogen can relieve aspirin injury in the stomach. Electrolyzed alkaline water itself can inhibit aspirin-induced gastric injury [29], and additionally, the alkaline load in water may prevent an increased urinary excretion of minerals like, calcium and magnesium, caused by the body’s acidity [30]. Dose-dependent inhibitory effects of hydrogen were observed in the stomach, but were not apparent in serum. The results suggest a dose–response effect exists when hydrogen interacts directly with the tissue, but a high dose of hydrogen may not increase the beneficial effects in target organs via blood transportation.

## Methods

### Ethics statement

Animal maintenance and experimental procedures were carried out in strict accordance with the Institutional Animal Care and Use Committee (IACUC) of Tsinghua University, which is a branch of Beijing Animal Care and Use Committee. All experiments were reviewed and approved by IACUC (permit number 12-LY-02), and all efforts were made to minimize suffering.

### Reagents

All solutions were prepared prior to use. Electrolyzed alkaline water was obtained from an alkaline ion water electrolyzer (TK7505, Panasonic, Japan), a commercial electrolyzer from Japan. The 8-OHdG and HEL ELISA kit were purchased from the Japan Institute for the Control of Aging (JalCA). Thiobarbituric acid was obtained from J&K Scientific Ltd; Hexadecyl trimethyl ammonium Bromide was acquired from Beijing River Dawn Biotechnology Co.,Ltd; and 3,3′,5,5′,-tetramethylbenzidine was obtained from Amresco, USA.

### Animals and gastric injury model

Male Sprague Dawley rats weighting 150-170 g were purchased from Beijing Vital River Laboratories, and housed at the Center of Biomedical Analysis, Tsinghua University. The experiment started when the rats reached 200-220 g in weight. Alkaline water with hydrogen was prepared every evening from facility tap water (pH 6.8, conductivity 40 μs/cm) and filled in aluminum bags. Two pH values, 8.5 and 9.5, were selected in this study. pH 8.5 and 9.5 are the up limits of drinking water standard in China and the standard for electrolyzer in Japan (please see Japan Industrial Standard, JIS T 2004:2005.), respectively. Alkaline water with pH 8.5 and 9.5 contained 0.07 ppm and 0.22 ppm hydrogen respectively during electrolysis (The electrolyzer is Panasonic TK7505 from Japan). Additionally, more hydrogen was dissolved in water with mixed hydrogen and nitrogen gas (2:8 and 7:3), which was filled into the aluminum bags at one atmospheric pressure, in order to yield 0.22 ppm and 0.84 ppm hydrogen water. Using nitrogen in the gas mixtures was a consideration for safety. Furthermore, the atmosphere contains 78% of nitrogen, which makes it unlikely a functional gas in our study. The concentration of hydrogen in water was measured by a portable dissolved hydrogen meter DH-35A (DKK-TOA Corporation, Japan). The hydrogen concentration was sustained for 24 h without detectable change.

Each animal was kept in a separate cage, and had free access to water at night from 6 pm to 9 am. The water intake and body weight were recorded daily for each rat. All rats were randomly distributed into 7 groups of 6 to 8 rats each, and given different drinking water (Table 1). Group A: pH 9.5, 0.84 ppm H_2_, B: pH 9.5, 0.22 ppm H_2_, C: pH 8.5, 0.84 ppm H_2_, D: pH 8.5, 0.22 ppm H_2_, E: pH 8.5, 0.07 ppm H_2_, F: pH 6.8, 0 ppm H_2_ (facility tap water), G: pH 6.8, 0 ppm H_2_ (facility tap water). The final hydrogen dose of each group was listed in Table 1. After two weeks of treatment, the animals were starved for 18 hours with hydrogen-rich water still available. The drinking water was removed one hour prior to the rats being dosed with 200 mg/kg of aspirin and 0.15 N HCl (8 mL/kg) together with 1% carboxymethyl cellulose sodium by intubation. Group G was starved, but not treated with aspirin-HCl as indicated in Table 1. After three hours, animals were anesthetized by urethane (1 mg/kg) and blood was taken from the abdominal aorta, following which they were sacrificed by exsanguination.

**Table 1:** Treatment groups and average body weight gain, daily water intake and presence of gastric injury

***Groups***	***pH***	***H***_***2 ***_ ***(ppm)***	***H***_***2 ***_ ***dose (μg/d/kg)***^**† **^ ***mean ± SE***	***Weight gain (g)***^**‡ **^ ***mean ± SE***	***Water intake (mL/d) mean ± SE***	***Injury***
A	9.5	0.84	80.6 ± 1.9	85.5 ± 6.0	25.6 ± 0.8	Yes
B	9.5	0.22	21.1 ± 1.6	81.9 ± 6.4	26.6 ± 2.5	Yes
C	8.5	0.84	80.6 ± 2.4	87.3 ± 5.0	25.9 ± 1.2	Yes
D	8.5	0.22	20.1 ± 0.8	78.5 ± 5.6	25.2 ± 1.4	Yes
E	8.5	0.07	6.8 ± 0.4	92.5 ± 3.0	26.9 ± 1.8	Yes
F	6.8	0	0	89.9 ± 3.5	27.2 ± 1.1	Yes
G	6.8	0	0	81.5 ± 7.0	26.3 ± 0.8	No

Each group was composed of 6–8 rats. ^**†**^The H_2_ dose for each rat was calculated by (hydrogen concentration x average daily water intake)/average body weight. ^**‡**^Weight gain was calculated by subtracting the body weight at the first day of treatment from the body weight before gastric injury. There is no significant difference between different groups.

### Evaluation of gastric mucosal lesions

After animals were euthanized, each stomach was removed, opened and washed with PBS. The eroded area on the surface of the hind stomach (gastric score) was measured under a dissecting microscope by a person without experiencing the dosing procedure. For histological evaluation, a piece of organ wall at the bottom region of the stomach was cut, paraffin embedded, Periodic Acid-Schiff (PAS) stained, haematoxylin counter-stained, and examined under light microscope. The gastric mucosa was scraped off from the rest stomach with a glass slide, and stored at −80°C.

### Myeloperoxidase (MPO) activity

Each gastric sample was ground by Teflon Potter-Elvehjem homogenizer in 500 μl 10 mM potassium phosphate buffer (pH 7.8) containing 30 mM KCl, 1% Phenylmethanesulfonyl fluoride and 5 mM EDTA, to get a homogenate. The homogenate was then centrifuged and the supernatant was used to detect the protein concentration. The pellet was re-homogenized in 500 μl of 0.05 M potassium phosphate buffer (pH 5.4) containing 0.5% hexadecyl trimethyl ammonium bromide, and then centrifuged. The 100 μl supernatant was then mixed with the same volume of 0.05 M potassium phosphate buffer (pH 5.4) containing 15 mM 3,3′,5,5′,-tetramethylbenzidine and 2% H_2_O_2_. MPO activity was detected by microplate reader at 630 nm every 15 seconds for 5 minutes, and it was expressed as units per mg protein. The MPO unit was defined as a change of absorbance (1.0/minute) at 630 nm in room temperature. The total protein concentration in the tissue homogenates was measured by the coomassie brilliant blue staining method.

### Malondialdehyde (MDA) relative concentration

The MDA generated in the gastric mucosa, as a product of lipid peroxidation, was detected by thiobarbituric acid reaction. Gastric mucosa was homogenized by Teflon Potter-Elvehjem homogenizer and ultrasonicated in 500 μl of 0.15 M KCl at 0°C to get a homogenate. The homogenate was separated into two tubes. One was used to measure protein concentration, while the other 150 μl was used to detect MDA. Protein was denatured by adding 150 μl of SDS, 150 μl of acetic acid, and 150 μl newly prepared 0.82% thiobarbituric acid solution. The mixture was placed in a boiling water bath for 45 min, then cooled and centrifuged. The supernatant was used to determine the relative MDA concentration by microplate reader at 532 nm.

### Measurement of serum 8-hydroxy-2′-deoxyguanosine (8-OHdG)

8-OHdG concentration is a biomarker of DNA damage and was detected by an ELISA kit (Cat. IM-KOGHS 040914E) from the Japan Institute for the Control of Aging. The test was conducted following the manufacturer’s instructions.

### Hexanoyl-lysine (HEL) adduct concentration

HEL is also a biomarker for oxidative stress. The HEL concentrations in serum were determined by an ELISA kit (Cat. KHL-700/E) from the Japan Institute for the Control of Aging. The test was performed following the manufacturer’s instructions.

### Statistical analysis

Results were presented as the mean ± standard error (SE), and the data were compared by Analysis of Variance (ANOVA) one way test by PASW Statistics 18. The differences were considered significant when the P-value was less than 0.05 by Tukey test.

## Results

### Drinking water consumption and gastric damage

As expected, the average volume of water consumption and the average weight gain per rat were similar across treatment groups, as shown in Table 1. These results imply that both the pH (8.5 and 9.5) and hydrogen concentration in drinking water (0.84 ppm, 0.22 ppm, and 0.07 ppm) did not affect the animals’ desire to consume water, and therefore the rats grew at the same rate. Thus, the gastric injury should not have been affected by potential differences in drinking behavior.

**Figure 1:**
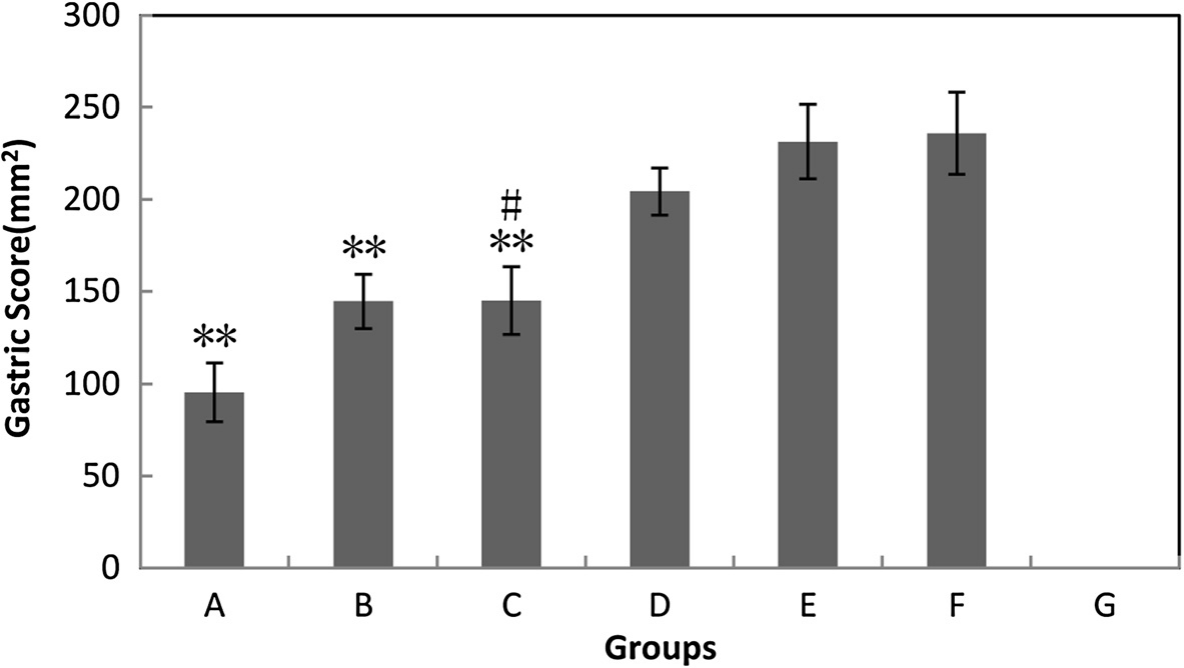
**Measurement of gastric score (the area of gastric erosions) of 7 groups.** Values are the mean ± SE for 6 to 8 animals. **: p < 0.01, compared with group F (neutral water control). #: p < 0.05, compared with group E (low hydrogen and low pH group).

Anatomical results showed that groups with high pH 9.5, high hydrogen concentration 0.84 ppm, or both (groups A, B and C), had significant inhibition of gastric damage compared with the group drinking facility tap water (group F) (Figure 1). And at the same pH, significant hydrogen-dose dependent inhibition was seen within groups C, D, and E. If the inhibitory effects and the hydrogen concentrations were compared, high positive correlation was found (Table 2). At the same hydrogen concentration, high pH also provided more inhibition (e.g. A and C, or B and D), though the effects were not statistically significant.

**Table 2:** Correlation between inhibition effects and hydrogen concentrations in pH 8.5 electrolyzed water

***Group***	***Erosion***^**†**^	***MPO***^**†**^	***MDA***^**†**^	***8-OHdG***^**‡**^
C: H_2_(6.8 μg/d/kg)	2.1%	1.6%	32.6%	−2.3%
D: H_2_(20.1 μg/d/kg)	13.6%	36.8%	36.8%	41.9%
E: H_2_(80.6 μg/d/kg)	38.6%	77.3%	61.9%	41.4%
***Pearson coefficient***	0.990	0.952	0.999	0.633

^**†**^Mucosal protective effect at each hydrogen concentration was calculated by (damage level in group F – damage level in group C or D or E)/(damage level in group F - damage level in group G).

^**‡**^In serum, since the gastric damage did not affect serum 8-OHdG level, the protective effect was calculated by (damage level in group F – damage level in group C or D or E)/damage level in group F.

**Figure 2:**
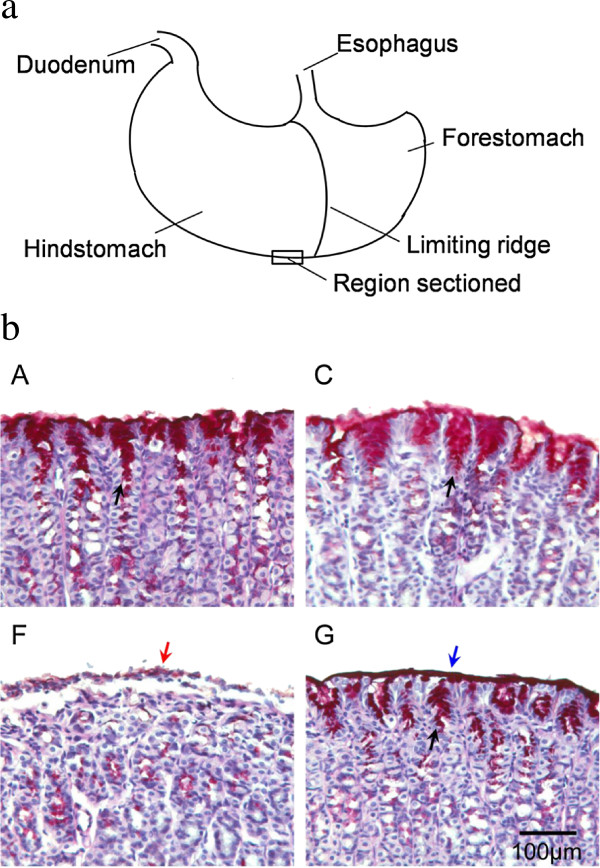
**The position of histological sample (a) and PAS staining of glandular stomach from groups A, C, F, and G (b).** Blue arrow indicates the mucus layer (red) on the surface of the inner wall, and red arrow points out the dying and detached cells. Black arrows show mucus producing cells (red). Magnification: 100. All the pictures show the representative phenotype of their corresponding samples.

Inhibitory effects were also evidenced through histological staining. Different regions of the stomach had different damage levels, which were not consistent within one dose group. We found that the bottom region of the stomach was usually evenly damaged by aspirin-HCl, and no bleeding erosion grove had been observed in that region. Therefore, histological tissues from that part were chosen for comparison as showed in Figure 2a. As expected, strong erosion was not observed in all samples (Figure 2b). Samples from group G had intact mucosal layer. Group F showed that the mucosal surface had detached cell debris as well as defective mucus production at many regions, which were pointed out in the Figure 2b. The samples from groups A and C had relatively intact mucus layer, and most mucus secretion cells were still functional compared to group F, which illustrated the inhibitory effects of hydrogen-rich electrolyzed water. Group B, D and E were also stained and checked under microscope, and they had middle levels of injury (Additional file 1: Figure S2). But the phenotype cannot be quantified.

### Hydrogen-rich electrolyzed water alleviated neutrophil-mediated inflammation and oxidative stress in stomach mucosa

**Figure 3:**
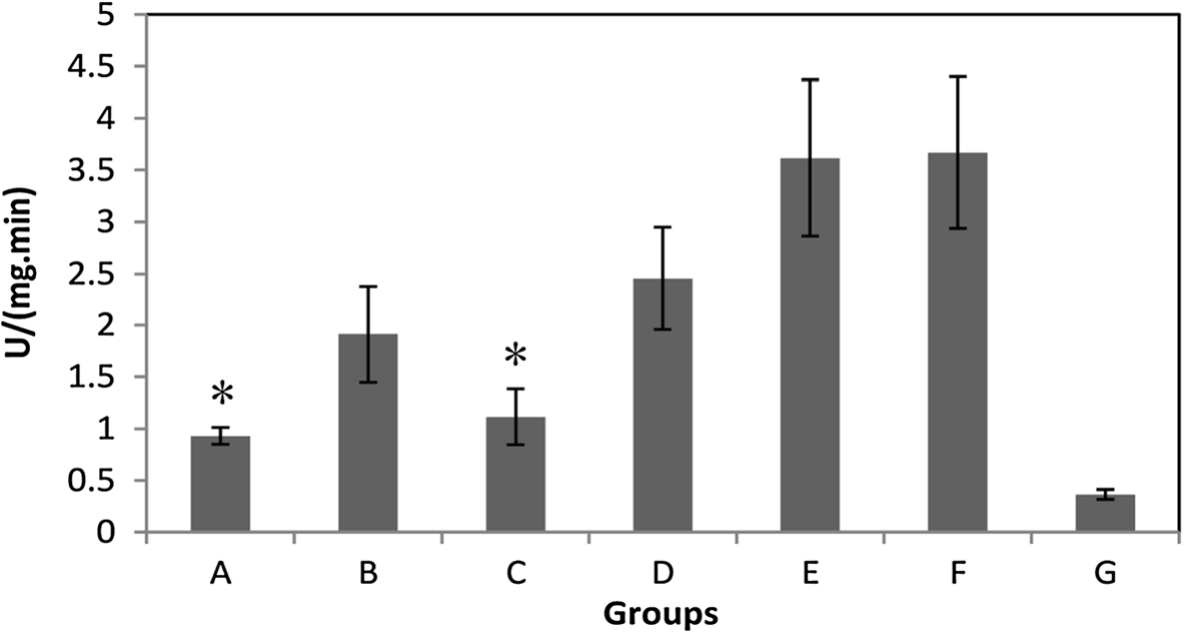
**The effect of hydrogen on MPO activity in gastric mucosa after injured by aspirin-HCl.** Values are the mean ± SE for 6 to 8 animals. *: p < 0.05, compared with groups E (low hydrogen and low pH group) or F (neutral water control).

Myeloperoxidase (MPO) activity from the mucosa was detected by thiobarbituric acid reaction and normalized with protein concentration for each sample. The activities were inhibited by pH 9.5 and 8.5 electrolyzed water in a hydrogen dose-dependent manner compared with group F (Figure 3). The inhibition levels were well correlated with hydrogen dose (Table 2).

**Figure 4:**
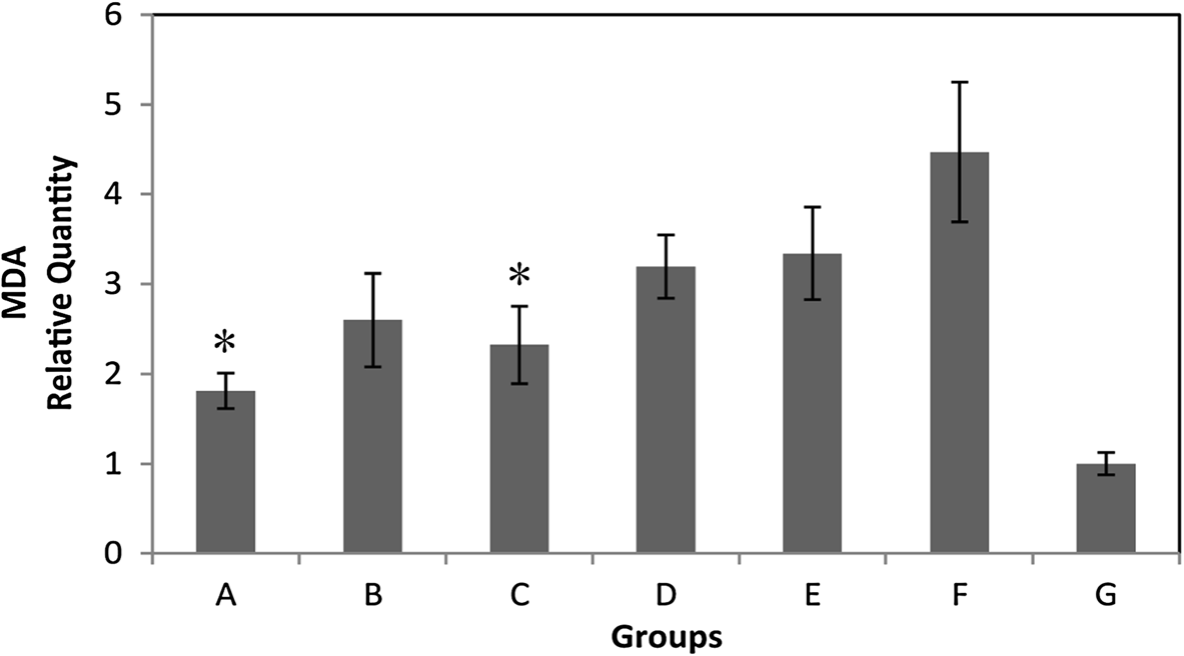
**MDA levels in gastric mucosa of 7 groups.** The quantity of MDA was normalized with the MDA level from group G (no injury control). Values are the mean ± SE for 6 to 8 animals. *: p < 0.05, compared with group F (neutral water control).

Malondialdehyde (MDA) is generated from reactive oxygen species (ROS), and as such, it is assayed in-vivo as a biomarker of oxidative stress. The mucosal MDA content in hydrogen-rich electrolyzed water treated groups showed significant reduction compared with the group drinking unaltered water (Figure 4). And the inhibition pattern is similar to the results of gastric score and MPO activity.

### Hydrogen-rich electrolyzed water reduced the level of 8-OHdG in serum

**Figure 5:**
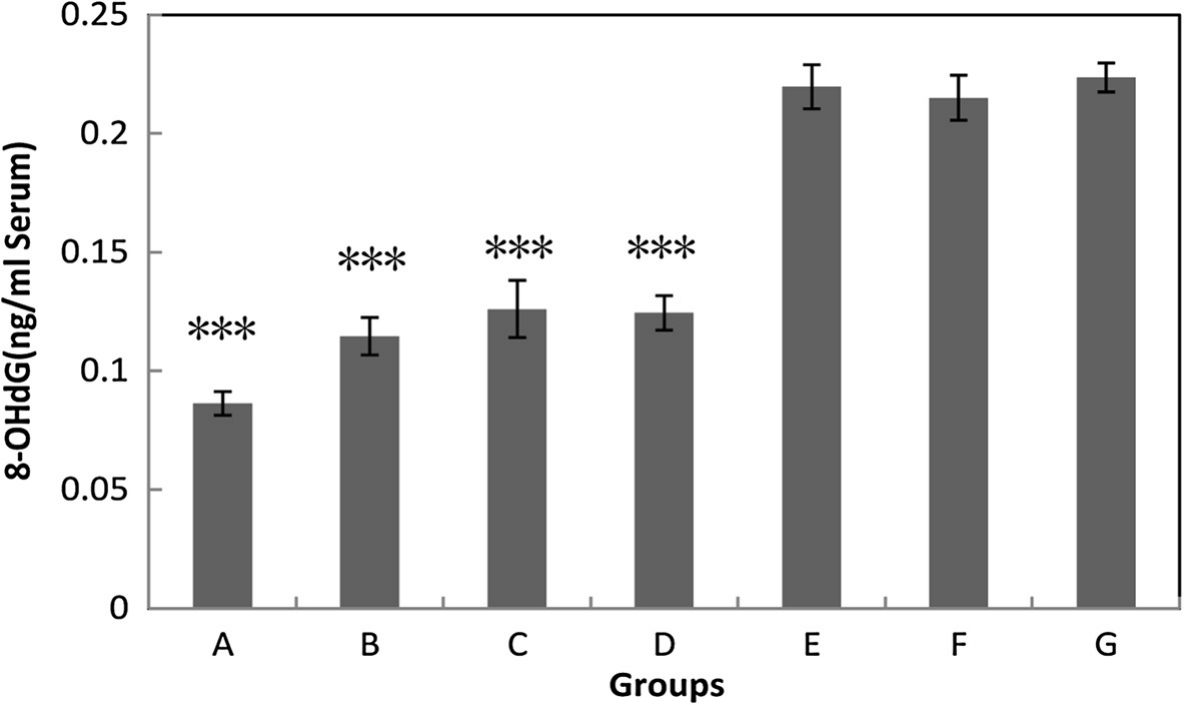
**The serum 8-OHdG level in different groups.** Values are the mean ± SE for 6 to 8 animals. ***: p < 0.001, compared with group E (low hydrogen and low pH group), F (neutral water control), or G (no injury control).

8-OHdG is one of the predominant forms of free-radical-induced DNA damage in nuclei and mitochondria, and therefore has been widely used as a biomarker for oxidative stress and carcinogenesis. The serum levels of 8-OHdG were significantly reduced in high hydrogen groups (hydrogen concentration larger than or equal to 0.22 ppm) compared with the tap water control group F (Figure 5). However, opposed to the other testing endpoints, reduction levels and hydrogen doses were not correlated in pH 8.5 groups, suggesting that a dose–response effect was not present for serum 8-OHdG (Table 2). The other different finding in 8-OHdG test was that the group G had similar level with group F, which might indicate both groups had background level of 8-OHdG. Another oxidative stress marker, HEL, which detects lipid peroxidation, was also tested in serum, but the results did not show significant differences among all the groups (Additional file 1: Figure S3).

## Discussion

As previously mentioned, one disconcerting issue in the application of hydrogen is the lack of dose–response effect. This study provided some evidence that hydrogen dose-dependent inhibitory effects can be observed in the aspirin-induced stomach injury model via electrolyzed alkaline water, and thus, the absence of an established relationship between dose and effect levels may be a consequence of delivery method, experimental design, and target organ.

In our study, we gave three different concentrations of hydrogen in alkaline water, and we observed obvious dose-dependent effects in the stomach mucosa, while most other reports only compared the effects with or without hydrogen [31-37]. All of them applied high hydrogen concentration from 0.8 ppm to 1.5 ppm, and all of them observed protective effects against different diseases or medical treatments. Although they did not give the exact drinking amount of the animals, we can estimate the hydrogen dose by its concentration since the animals were always free to access water and hydrogen did not change their drinking behavior. Only one study applied two different hydrogen concentrations (0.08 and 1.5 ppm) in the treatment of mouse model of Parkinson’s disease. However, their results suggested both dosages had a similar function in alleviating the progress of neurodegeneration [22]. Another main difference between our and previous studies is that the hydrogen was delivered through drinking water directly into the stomach, instead of being transported by the blood to the target organs [17,18]. Water can be absorbed in the stomach, and we believe that hydrogen concentrations in the mucosal cells of the stomach vary depending on the hydrogen concentration in the water. However, for other organs this may not be the case, since hydrogen gets into the blood first, and then is transported to all organs throughout the body. Hydrogen pharmacokinetics is not fully understood; yet, a recent study showed that hepatic glycogen can accumulate hydrogen from drinking water [38]. This study not only revealed one of the reasons why consumption of even a small amount of hydrogen over a short time span efficiently improves various disease models, but also suggested that hydrogen concentration can be buffered in the blood. We regard this glycogen buffering effect as one of the reasons for the absence of dose–response phenomena in many other studies. In this study, the 8-OHdG levels in serum changed without dose-dependent effect which supports this hypothesis.

Although groups with high hydrogen concentration showed significantly lower serum 8-OHdG level compared with the group drinking facility tap water after gastric injury, it was surprising that group G which had not been injured with aspirin-HCl, had nearly the same level of 8-OHdG as the injured group F. This might mean that little DNA damage occurred in the stomach, or that the damaged stomach mucosa did not release 8-OHdG into blood during the 3 hours post injury. The hydrogen may have reduced the background level of 8-OHdG in serum. We think this could be the same reason for unchanged HEL level in the serum. Oxidative damage in the stomach did not affect the blood very much, and the background level of HEL is too low to be detected by our kit.

Although many endpoints were tested in our injury model, gastric score, MPO activity and MDA quantity are the best. The gastric erosion is obvious under dissection microscope 3 hours after aspirin-HCl treatment, and the erosion area can be calculated by using a micro-ruler. However, the severity of erosion had not been considered as most previous studies did. Some erosion areas were irregular white spots, while bleeding could be seen on other regions. There is no rule for setting the weights for different erosion severities. We think it could be a possible improvement, which can be done for this injury model in the future.

MPO is most abundantly expressed in neutrophil granulocytes. Substantial evidences have suggested that neutrophil-mediated inflammation is involved in the development of aspirin-induced gastric injury [39-42]. Naito et al. [29] found that MPO activity increased in the gastric mucosa after 1 h aspirin treatment and persisted at 3 h, and could be reduced by pH 10 electrolyzed water. The inflammation is usually induced by the dying cells in the tissue. It is difficult to directly detect the quantity of dying cells with mucosa, since some of them are already broken to pieces. Thus, examining the living inflammatory cells is a wise choice.

Oxidative stress is one of the major effects caused by aspirin treatment in the stomach. And hydrogen has been reported to relieve oxidative stress in many tissues [17,18]. MDA is one of the best known biomarkers for oxidative stress. It comes from the degradation of polyunsaturated lipid by reactive oxygen species. It is one of the many reactive electrophile species which can form covalent protein adducts referred to as advanced lipoxidation end-products (ALE). Before we saw the results, we were afraid that MDA is too reactive to be detected in the samples which had been stored in the fridge for several weeks. Fortunately, the results showed a consistent reduction of MDA with hydrogen dose, which demonstrates it is a reliable and stable endpoint in aspirin-HCl injury model.

The TNF-α expression was reported to be significantly enhanced in the aspirin-HCl injured stomach and serum [29]. And drinking electrolyzed alkaline water could decrease TNF-α at both protein and mRNA level. We also tested the TNF-α mRNA level in the stomach and the TNF-α mRNA did increase in the injured stomach. However, we did not find significant change between different treated groups (Additional file 1: Figure S4). It might be due to the time point of harvesting the tissue sample. TNF-α is an early responder to the gastric injury and can induce apoptosis of gastric epithelial cells as well as endothelial cells [43-45]. The significant inhibition of TNF-α mRNA expression by electrolyzed water treatment can be observed at 1 hour after aspirin treatment [29], but not at 3 hours as communicated with the author of this previous work. At 1 hour after the aspirin-HCl treatment, the erosion can not be easily observed, which means the mucosal layer is still relatively intact. At that time, any piece of the stomach may have similar TNF-α mRNA level. However, when some erosions formed at 3 hours, different pieces of the stomach wall may have different TNF-α mRNA levels, since the erosion parts are relatively more heavily damaged. We consider that it is better to take the whole stomach for TNF-α mRNA quantification other than a piece of a stomach wall, but it will need a double number of animals.

Another point in question is the mechanism of hydrogen-rich electrolyzed alkaline water induced damage inhibitory effects. Early work already demonstrated that the inhibition of gastric damage is indirectly induced by continuous electrolyzed alkaline water treatment, but not by direct interaction of electrolyzed alkaline water and aspirin [29]. Furthermore, the inhibitory effects of electrolyzed alkaline water were not caused by reducing the gastric acidity, which might affect absorption of aspirin [29]. The hydrogen in electrolyzed alkaline water was suggested as an active molecular. Ohsawa et al. found the direct reaction between hydrogen and hydroxyl radical and peroxynitrite [7]. However, this mechanism does not fit our observations because the drinking water was removed one hour before the gastric injury took place. According to two previous reports, hydrogen could have been exhaled out within 10 minutes after drinking [17] and the hydrogen concentration in liver and kidney should have dropped to background level within 40 minutes after one dose of injection [46]. Therefore, it is unlikely that the inhibitory effects resulted from the reductive reaction by molecular hydrogen. Alternatively, a more favored mechanism would involve hydrogen acting as a signaling molecule that alters gene expression in the cell, therefore improving the cell’s anti-damage state. The expression alterations of many genes and proteins caused by hydrogen, such as pro-inflammatory cytokines, nuclear factors, and caspases, have been reported in other disease models [17]. However, further gene regulation profile study is necessary to answer the question in the stomach injury model.

## Conclusions

Overall, this study provided evidences that hydrogen can inhibit aspirin-HCl-induced stomach injury in a dose-dependent manner. The lack of dose–response effect observed in other studies might be a result from a blood glycogen buffering effect. Drinking hydrogen-rich water may protect healthy individual from gastric damage caused by oxidative stress.

## Abbreviations

MPO: Myeloperoxidase; MDA: Malondialdehyde; 8-OHdG: 8-hydroxy-2′-deoxyguanosine; HEL: Hexanoyl-Lysine.

## Competing interests

The authors declare that they have no competing interests.

## Authors’ contributions

XJL, SGD and HLY performed the research, XJL, SGD analyzed the data, LY, LWJ and VN wrote the paper. All authors read and approved the final manuscript.

## Pre-publication history

The pre-publication history for this paper can be accessed here:

http://www.biomedcentral.com/1472-6882/14/81/prepub

## Supplementary Material

Additional file 1: Figure S1-S4 The daily body weight changes, histology of group B, D, and E, serum HEL levels, and relative abundance of TNF-a mRNA. Click here for file
