# Supplementary material for: Dose-dependent inhibition of gastric injury by hydrogen in alkaline electrolyzed drinking water
Source: BMC Complement Altern Med. 2014 Mar 3;14:81. doi: 10.1186/1472-6882-14-81 (PMC3944674; doi:10.1186/1472-6882-14-81)
Supplement: Additional file 1: Figure S1-S4 — The daily body weight changes, histology of group B, D, and E, serum HEL levels, and relative abundance of TNF-a mRNA. [file 1472-6882-14-81-S1.doc]

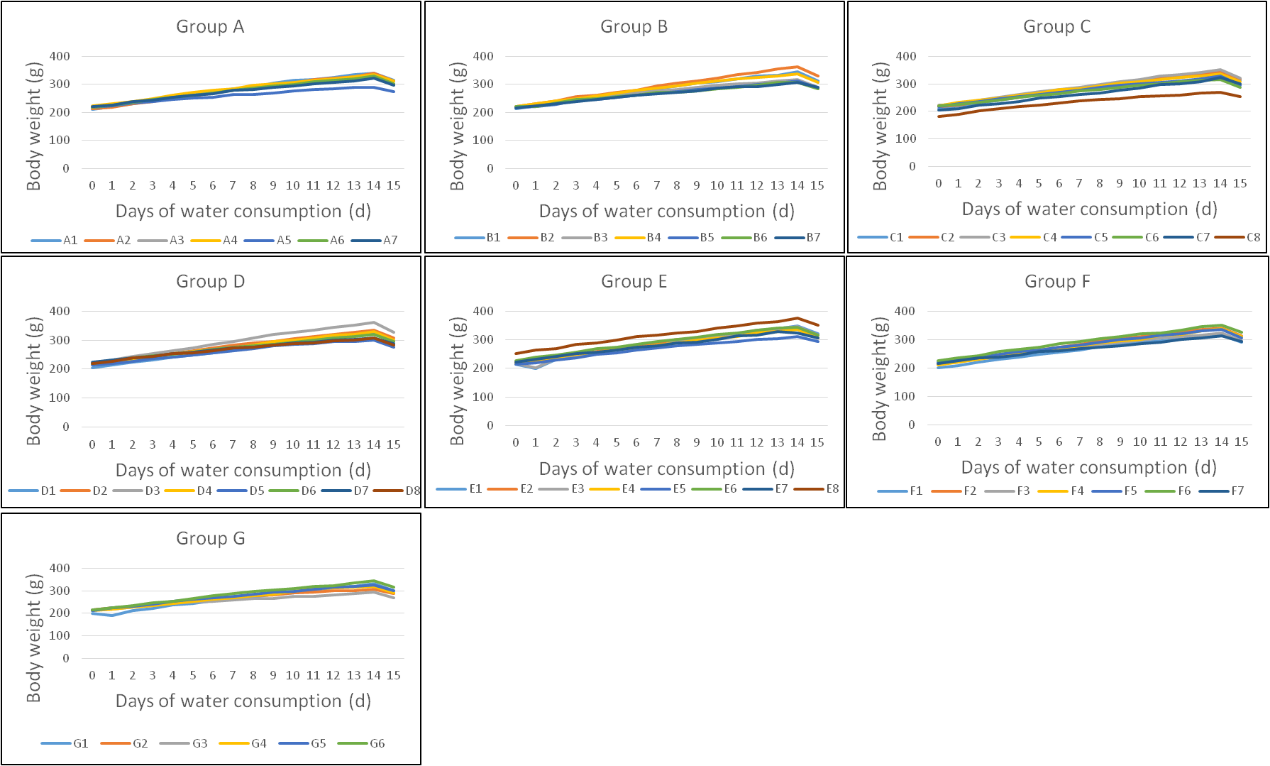


**Figure S1 Daily body weight changes of each rat in 7 groups. Every color in each group represents one rat. The weight loss on final day was due the 18 hours starving.**


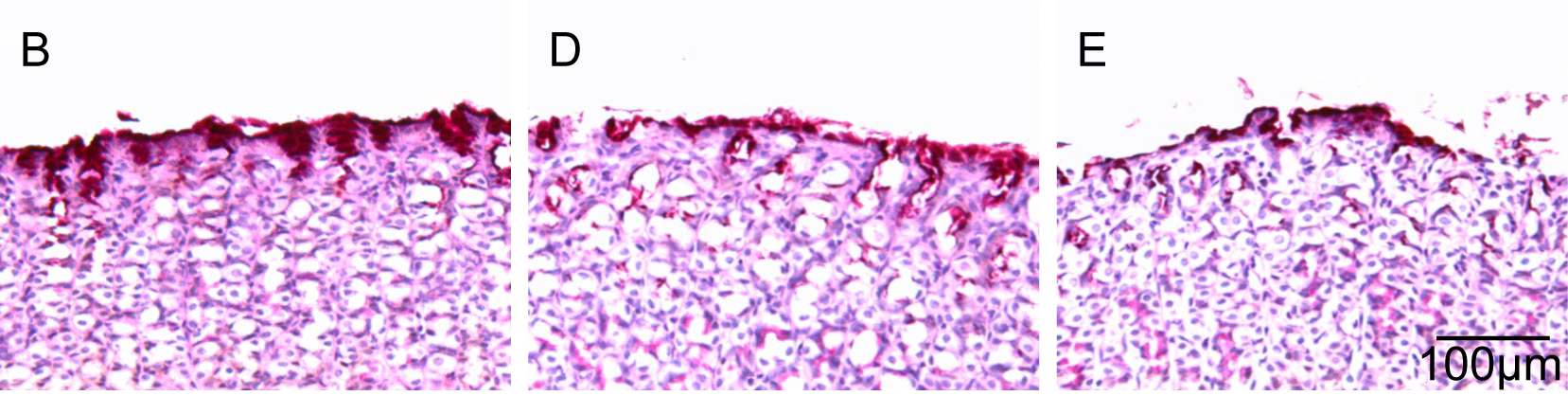


**Figure S2 PAS staining of hindstomach from group B, D, and E.**


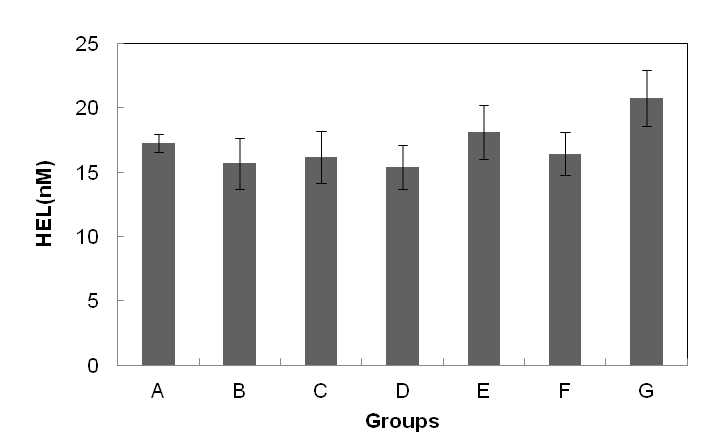


**Figure S3 The serum HEL level in different groups. Values are the mean±SE from 6 to 8 animals.**


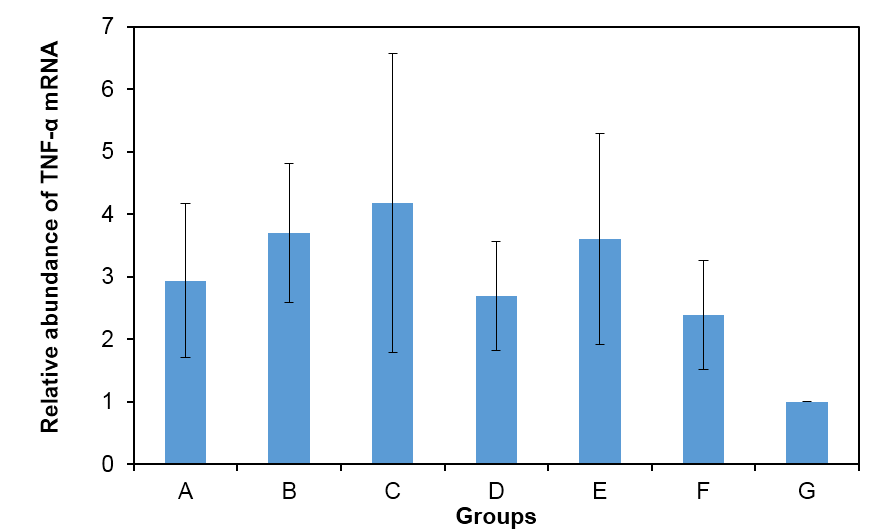


**Figure S4 Relative abundance of TNF-α mRNA in the hindstomach of 7 groups. The level of TNF-α mRNA in group G was normalized to 1.**
